# Construction Notes

**Published:** 1905-04-22

**Authors:** 


### CONSTRUCTION NOTES.

Plans have been drawn out for a new small-pox hospital for Chester-le-Street.

A small building committee has been formed to consider at an early date the provision of a new operation theatre for the Royal Portsmouth, Portsea, and Gosport Hospital.

The Committee of the Consumption Sanatorium at Knightwick are anxious to make further additions and alterations to the institution. It is urgent to provide further accommodation.

It is proposed to erect a central hospital in the Highlands near Kingussie. The scheme has been under consideration for some years already. The total cost is estimated at £3,472.

It is proposed to extend the Falkirk Infirmary by building a new administrative block, an additional ward of six beds, and a new operating and anæsthetic rooms, at an estimated cost of £2,300.

Amongst the many recent improvements at the Leicester Infirmary is a rearrangement of the steam plant, the warming of the whole building on the vacuum system, and the service of hot water throughout the infirmary. A new boiler, 30 feet by 7 feet 6 inches, has been fixed, and the old one, 24 feet by 7 feet, refixed for emergency. Steam is taken down to the pump-room through high-pressure steel mains into a distributing drum, being thence directed for all purposes of the institution, including supplies to the disinfector, the destructor, the sterilisers, the laundry engine, the washing machinery, and the kitchen requisites. All the mains for these purposes are trapped and drained, and the water of condensation and vapour, which would otherwise be wasted, is returned to the pump-room, and, by means of the "Nuconomiser" system, reapplied in such a manner as to provide all the heat necessary for the hot-water service, and, in addition, to feed the boiler with water at 205° F., thus effecting a substantial saving in fuel. It should be added that the exhaust steam from the pumps and the laundry engine is also utilised in this way, after passing through a grease separator.

From the pump-room two large subways, 6 feet 6 inches by 5 feet, connect the infirmary buildings and the new out-patients' block, and convey the steam mains on adjustable brackets, which allow freedom of access and supervision. The heating of the infirmary has been rearranged and adapted from a hot-water system to the vacuum system of steam, as much as 10-inches of vacuum being maintained by a large vacuum pump, from which also the water of condensation is collected and utilised for feeding the boiler at the high temperature already indicated.

CHRISTIA TISSUE.

The address of the company is T. Christy and Co., 4 Old Swan Lane, E.C.

